# Chu-Style Lacquerware Dataset: A Dataset for Digital Preservation and Inheritance of Chu-Style Lacquerware

**DOI:** 10.3390/s25175558

**Published:** 2025-09-05

**Authors:** Haoming Bi, Yelei Chen, Chanjuan Chen, Lei Shu

**Affiliations:** 1 Digital Art Industry Institute, Hubei University of Technology, Wuhan 430068, China; 2 NAU-Lincoln Joint Research Center of Intelligent Engineering, Nanjing Agricultural University, Nanjing 210031, China

**Keywords:** digital preservation and inheritance of traditional Chinese crafts, Chu-style lacquerware, artificial intelligence-generated model, low-rank adaptation training technique, intangible cultural heritage preservation

## Abstract

The Chu-style lacquerware (CSL) dataset is a digital resource specifically developed for the digital preservation and inheritance of Chu-style lacquerware, which constitutes an important component of global intangible handicraft heritage. The dataset systematically integrates on-site photographic images from the Hubei Provincial Museum and official digital resources from the same institution, comprising 582 high-resolution images of Chu-style lacquerware, 72 videos of artifacts, and 37 images of traditional Chinese patterns. It comprehensively demonstrates the artistic characteristics of Chu-style lacquerware and provides support for academic research and cultural dissemination. The construction process of the dataset includes data screening, image standardization, Photoshop-based editing and adjustment, image inpainting, and image annotation. Based on this dataset, this study employs the Low-Rank Adaptation (LoRA) technique to train three core models and five style models, and systematically verifies the usability of the CSL dataset from five aspects. Experimental results show that the CSL dataset not only improves the accuracy and detail restoration of Artificial Intelligence (AI)-generated images of Chu-style lacquerware, but also optimizes the generative effect of innovative patterns, thereby validating its application value. This study represents the first dedicated dataset developed for AI generative models of Chu-style lacquerware. It not only provides a new technological pathway for the digital preservation and inheritance of cultural heritage, but also supports interdisciplinary research in archeology, art history, and cultural communication, highlighting the importance of cross-disciplinary collaboration in safeguarding and transmitting Intangible Cultural Heritage (ICH).

**Dataset:** The dataset has been released on Hugging Face as open source, available at https://huggingface.co/datasets/TenFate/CSL_Dataset (accessed on 2 September 2025). This dataset is for research purposes only and may not be used for commercial purposes in any form.

**Dataset License:** apache-2.0

## 1. Introduction

The development of Chinese lacquerware is recognized as a unique symbol of civilization, with a history dating back to the late Neolithic Hemudu culture (circa 4000 BCE) [1]. As an important branch within the Chinese lacquerware lineage, Chu-style lacquerware developed distinctive regional characteristics during the Warring States period (475–221 BCE). The craftsmanship of Chu-style lacquerware includes three major categories: (1) tenon and mortise woodcarving, (2) gold-lacquered bowls and trays, (3) the use of gourd and bone molds. These techniques employ layered assembly, colorful lacquer mixing, and other processes, creating a unique artistic style characterized by “three parts carving, seven parts coloring.” The esthetic qualities of Chu-style lacquerware are showcased through line esthetics, color systems, and spatial composition, while the natural worship patterns, geometric abstractions, and social and mythological patterns systematically reflect the unique romantic esthetics and rich spiritual connotations of Jingchu culture. The curved dynamic forms, the black, red, and gold color scheme, and the phoenix, bird, and mythical beast patterns of Chu-style lacquerware stand out when compared to lacquerware from other regions. Cultural relics, e.g., the Tiger Seat Bird Rack Drum and the Painted Chariot and Horse Figure unearthed in Jingzhou, Hubei, demonstrate the extensive use of Chu-style lacquerware in ceremonial and musical instruments. In 2011, the “Chu-style Lacquerware Lacquering Technique” was included in the national list of ICH.

As a traditional craft with distinctly Eastern cultural characteristics, Chu-style lacquerware embodies thousands of years of historical accumulation and carries profound cultural and artistic value. However, its transmission faces numerous difficulties and challenges, necessitating the use of digital technologies to support its preservation and development. Chu-style lacquerware is subject to the dual erosion of time and environment: many artifacts, due to their age, inevitably suffer natural degradation, while environmental factors, e.g., fluctuations in humidity and temperature and harmful substances in soil also cause varying degrees of damage. In the face of the gradual decline of this precious cultural heritage, digital preservation has become an urgent mission [2,3]. Compared with traditional conservation techniques, digital preservation’s most significant advantage lies in its non-contact approach. By generating digital archives, this method not only overcomes the limitations of physical space and artifact fragility but also greatly expands opportunities for public engagement in heritage protection [4,5]. Digital archives comprehensively record the current state of artifacts and establish a permanent data foundation for subsequent research, fundamentally avoiding the secondary damage common in traditional conservation processes and achieving truly non-invasive preservation [6].

In recent years, advances in artificial intelligence generation technologies have provided a new perspective for the digital preservation of Chu-style lacquerware. However, these technologies still face certain challenges. Mainstream generative models, e.g., Stable Diffusion XL [7], DeepFloyd IF [8,9], and DALL-E 2 [10] are generally pretrained on datasets dominated by Western cultural visual elements and English textual descriptions, resulting in a degree of “cultural centrality” misalignment. This misalignment manifests in the models’ inability to accurately capture and reproduce the specific cultural symbols and unique decorative techniques embodied in Chu-style lacquerware, leading to inaccurate outputs and severely limiting their applicability in its digital preservation.

To this end, this study is dedicated to constructing a high-quality CSL dataset. The objective of this dataset is to provide a solid data foundation for artificial intelligence generative models and to address the adaptability problem between AI generative models and Chu-style lacquerware images. The CSL dataset systematically integrates the official digital resources of the Hubei Provincial Museum, multi-device field-collected samples, and online resources, gathering high-quality, multi-perspective Chu-style lacquerware data. This ensures both the dataset’s integrity and the detailed representation of patterns. A combined annotation approach of automatic labeling and manual verification was adopted, producing high-quality text descriptions for each image. These descriptions accurately capture the morphological characteristics of the artifacts and the detailed features of their patterns. This study employs LoRA [11] technology to validate the dataset. At the research level, this method serves as one of the essential technical approaches for the verification of the CSL dataset; at the public level, it provides users with a pathway to engage with and understand Chu-style lacquerware through the CSL dataset. Through this approach, AI generative models can better learn the artistic characteristics of Chu-style lacquerware, thereby generating images more accurately aligned with its style. The purpose of this study is not only to offer systematic technical support for the digital preservation and transmission of Chu-style lacquerware, but also to provide a theoretical framework and dataset reference for subsequent research, while enabling the public to appreciate its cultural significance.

The LoRA method was proposed by Edward Hu et al. at ICLR in 2022 to address the prohibitive cost of full fine-tuning large pre-trained language models on downstream tasks. The core idea of LoRA is to freeze the parameters of the pre-trained model while inserting trainable low-rank decomposition matrices, enabling efficient parameter updates. This greatly reduces storage and computational requirements while maintaining, or even surpassing, the performance of full fine-tuning. In numerous experiments on models, e.g., RoBERTa, DeBERTa, GPT-2, and even GPT-3, LoRA achieved performance comparable to or better than full fine-tuning on downstream tasks. Since this method can achieve results equivalent to full fine-tuning by training only a small number of additional parameters, with virtually no increase in inference overhead, it significantly lowers the GPU memory and computational cost of training. For this reason, this study adopts LoRA for model fine-tuning.

The main contributions of this study are summarized in three aspects:

- Dataset construction: For the first time, this study establishes a high-quality image dataset of Chu-style lacquerware, providing a solid data foundation for AI models to learn Chu-style lacquerware features and to understand its cultural significance.
- Model optimization: Based on this dataset, three core models and five style models were successfully trained, enhancing both the accuracy and artistic expressiveness of Chu-style lacquerware image generation.
- Interdisciplinary value: This research provides a reusable technical pathway for the digital preservation and inheritance of intangible cultural heritage, and introduces new avenues for the public dissemination and educational promotion of Chu-style lacquerware culture.

This paper is organized into six chapters: Section 1 discusses the research background and motivation for the digital preservation of Chu-style lacquerware. Section 2 reviews related work in the field. Section 3 presents the composition, data sources, and scale of the CSL dataset. Section 4 introduces the dataset construction methods and model training scheme. Section 5 outlines the evaluation metrics, presents comparative experimental results, and discusses the findings. Section 6 summarizes the overall work, explores its academic value and practical significance, and provides an outlook on potential applications and future research directions.

## 2. Related Work

In this chapter, the relevant exploration can be divided into three directions: (1) the preservation approaches of ICH handicrafts, e.g., lacquerware; (2) the current state of research in the field of lacquerware; (3) the construction of cultural relic datasets through digital methods. This chapter develops its discussion around these three aspects, examining practices related to the preservation of cultural relics, especially ICH handicrafts, e.g., lacquerware, reviewing relevant research in the lacquerware field, and summarizing the existing progress in the construction of cultural relic datasets. It also highlights the insufficiency of resources related to Chu-style lacquerware, thereby underscoring the significance of constructing a CSL dataset in this study.

### 2.1. Protection Pathways for Intangible Cultural Heritage Handicrafts

In the study of cultural relic preservation, the protection and inheritance of ICH handicrafts hold significant importance. Cultural relics, e.g., lacquerware, as highly representative artifacts of ancient China, not only embody profound historical and cultural connotations but, due to the characteristics of their materials, are also highly susceptible to environmental damage. Therefore, while emphasizing the transmission of traditional craftsmanship, it is also necessary to introduce modern technologies to explore sustainable preservation solutions in this field. Marek Milosz et al. (2022) examined the overall use of 3D digital technologies in cultural relic preservation [12]; Huafeng Quan et al. (2024), taking the batik culture of the Miao ethnic group in Guizhou Province, China as a case study, investigated the application of knowledge graphs, natural language processing, and deep learning techniques in the promotion and preservation of cultural relics [13]; Ying Huang et al. (2024) proposed a distributed optical fiber sensing technology for corrosion monitoring, which supports the monitoring of material corrosion and damage characteristics in complex environments, thereby providing technical support for preventive preservation of cultural relics [14]; Zishan Xu et al. (2024), through the construction of the MuralDH dataset, developed valuable digital resources for the preservation and restoration of Dunhuang murals [15]. Table 1 presents descriptions of protection pathways for ICH handicrafts.

### 2.2. Current Lacquerware Research

This study reviewed ten representative scientific publications in the field of lacquerware published between 2020 and 2025. Liping Zheng et al. (2020) employed multiple scientific analytical methods to investigate the materials and production techniques of the “Chun Rong Xuan Mao” birthday plaque, which holds significant historical value for studying the decorative techniques of ancient lacquerware [16]; Yingchun Fu et al. (2020) examined two types of lacquerware unearthed from the Chu tombs at Jiuliandun, Hubei, China, dating to the Warring States period, revealing their multilayer lacquering techniques [17]; Meng Wu et al. (2021) analyzed the lacquer and pigments of polychrome lacquerware excavated from the tomb of Marquis Yi of Zeng, also dating to the early Warring States period [18]. Xin Wang et al. (2021) conducted a comprehensive study on the materials, structure, and lacquering techniques of a lacquered wooden coffin from the Qing dynasty’s Eastern Royal Tombs [19]; Bin Han et al. (2022) applied multidisciplinary methods including SEM-EDS and Py-GC/MS to analyze a Warring States lacquer scabbard unearthed at the Lijiaba site [20]; Kai Wang et al. (2022) studied ancient lacquer films excavated from the Western Han tomb M6 at Dongshan, Taiyuan, Shanxi Province, China, further demonstrating the pH-dependent warping behavior of ancient lacquer films [21]; Danfeng Hu et al. (2023) investigated lacquerware fragments recovered from the “Nanhai I” shipwreck, applying techniques, e.g., XRD, SEM-EDS, FT-IR, and confocal Raman spectroscopy to analyze lacquering processes in terms of layering, lacquer film composition, and substrate structure [22]; Zisang Gong et al. (2024) explored the manufacturing process of painted gold foil on Western Han lacquerware unearthed at the Jinyang ancient city site, providing guidance for the preservation of gold-foil-decorated lacquerware [23]; Zhanyun Zhu et al. (2025) studied a lacquer screen from the tomb of Sima Jinlong of the Northern Wei dynasty using multidisciplinary methods, revealing its complex structure and offering valuable insights for the preservation and restoration of lacquerware relics [24]; Jiake Chen et al. (2025) investigated a group of Han dynasty lacquerware unearthed from tomb M9 at Guishan, Luoping, Yunnan Province, China, analyzing lacquering techniques and raw materials using pyrolysis-gas chromatography–mass spectrometry and X-ray fluorescence spectroscopy [25]. Table 2 describes the current state of lacquerware research.

### 2.3. Research on Cultural Relics Datasets

In the existing literature on datasets, no research has yet been identified regarding the construction of a Chu-style lacquerware dataset. The HeluoMusicalRelicals_Xia-Qing system compiled and studied 102 musical artifacts from the Xia to the Qing dynasties unearthed in Luoyang, Henan, China, integrating artifact images and analytical texts into a single document file [26]. Hanyu Xiang et al. employed laser scanning and close-range photogrammetry to achieve millimeter-level precision in the three-dimensional digitization of Cave 13 of the Yungang Grottoes, constructing a dataset comprising 283,400 images, 433 stations of laser scan point clouds, and corresponding 3D model files [27]. EmbroideryCulturalRelics contains 260 embroidery artifacts dating from the Qing dynasty to the Republic of China, accompanied by high-resolution photographs and metadata tables that include artifact numbers, names, thumbnails, types, and periods [28]. DeepJiandu is a publicly available dataset dedicated to character detection and recognition of bamboo slips (Jiandu), consisting of 7416 infrared images covering 2242 categories [29]. Art_GenEvalGPT is a synthetic art dialog dataset generated using ChatGPT 3.5, which includes 13,870 dialog pairs, covering 799 artworks, 378 artists, and 26 art styles, thereby enriching the dataset resources in the domain of art-related dialogs [30]. Compared with mature databases on musical artifacts, grotto digitization, embroidery heritage, bamboo slip character archives, and art dialogs, the digital resources for Chu-style lacquerware remain underdeveloped. This gap highlights the innovative significance of constructing a CSL dataset in this study.

The construction of the CSL dataset enriches the digital resources available for the preservation and transmission of Chu-style lacquerware, ensuring the continuation of its cultural lineage while validating a technical pathway for its digital protection and inheritance. With the core and style models trained on the CSL dataset, researchers can directly obtain digital samples aligned with the esthetic paradigms of Chu-style lacquerware. This study further establishes a complete workflow, ranging from data collection and processing to model training. The framework can be extended to other forms of ICH handicrafts, e.g., bronzeware, porcelain, paper cutting, and embroidery, thereby providing a valuable reference for interdisciplinary research.

## 3. Dataset Description

### 3.1. Dataset Introduction

**Figure 1:**
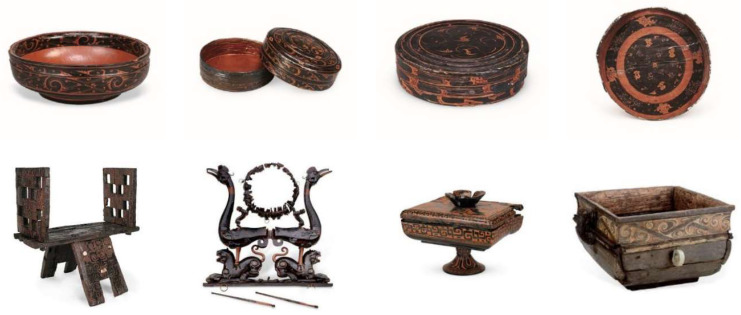
Data from online resources of Hubei Provincial Museum.

**Figure 2:**
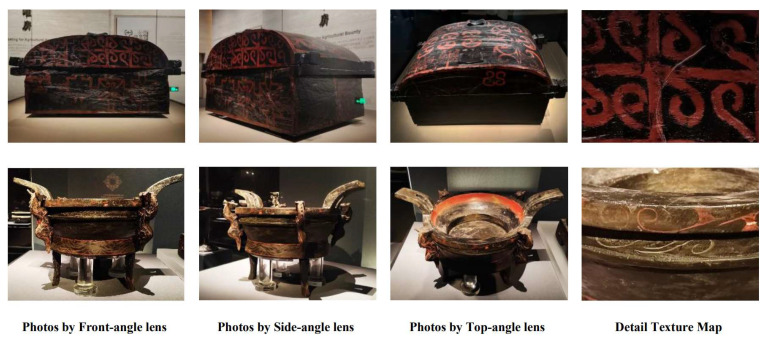
Offline-captured three-view images and detailed patterns of Chu-style lacquerware.

**Figure 3:**
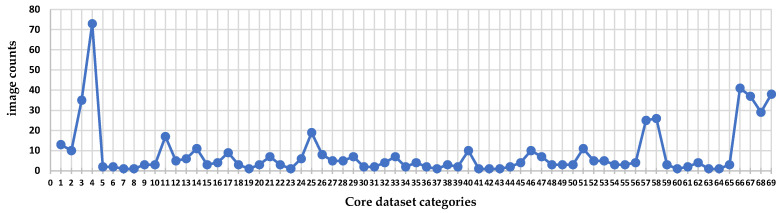
Statistical distribution of image counts across 69 categories in the core dataset.

The CSL dataset constructed in this study is designed to provide data support for the digital preservation and inheritance of intangible handicraft heritage. The dataset consists of three categories: (1) the core dataset, (2) the style dataset, (3) the video dataset. The dataset is divided into 69 classes, containing a total of 582 images, each with a corresponding annotated text description. The image resolution is 4096 × 3072. The style dataset is divided into 5 classes, comprising 37 images with a resolution of 1024 × 1024. The video dataset is divided into 35 classes, containing 72 videos with a resolution of 1920 × 1080. Table 3 presents an overview of the CSL dataset. Figure 1 shows images obtained from the official website of the Hubei Provincial Museum. Figure 2 displays three-view images and detailed patterns of Chu-style lacquerware captured on-site. Figure 3 illustrates the distribution of the number of images across the 69 categories in the core dataset. In this study, only the core dataset and the style dataset of the CSL were processed, while the video dataset was not addressed. The following subsection will focus on the processing methods of the core dataset. Since the processing methods of the style dataset are similar to those of the core dataset, they will not be elaborated upon in the subsequent discussion.

In this study, the CSL dataset was compared with other datasets in related fields. As shown in Table 4, the construction of the CSL dataset provides an important supplement to the training data of artificial intelligence for Chu-style lacquerware. Its core characteristics include high-resolution image data, a combined annotation approach integrating automatic labeling and manual verification, and annotation files that embody the artistic features of Chu-style lacquerware.

### 3.2. Data Collection

In terms of data collection, to construct a high-quality and highly usable Chu-style lacquerware dataset, this study employed two channels: online resource acquisition and offline field photography, ensuring both comprehensiveness and reliability of the data. The online resources were mainly obtained from the official website of the Hubei Provincial Museum. Their advantages lie in strong systematicity and wide coverage, providing a relatively complete reflection of the classification system of Chu-style lacquerware. However, some images suffer from low resolution, blurred artifact edges, and insufficient color information, making them unsuitable for training purposes. To compensate for these shortcomings, field investigations were conducted at the Hubei Provincial Museum, where target artifacts were photographed from multiple angles using devices, e.g., smartphones and Digital Single-Lens Reflex Cameras. The advantage of this approach is that it ensures comprehensive coverage of artifact categories while simultaneously expanding the sample size within individual categories, thereby meeting both the quantity and quality requirements of cultural heritage image data for deep learning models [31]. The disadvantages, however, include potential noise and glare in some images due to exhibition hall lighting, reflections, and shooting angle limitations. Table 5 presents a comparison of the advantages and disadvantages of data collection methods for the core dataset. The style dataset was primarily compiled from online resources, consisting of images of traditional Chinese patterns. These data were sourced from the social media platform Xiaohongshu and did not include offline acquisitions. The video dataset, on the other hand, was entirely obtained through on-site filming at the Hubei Provincial Museum, without the use of online resources.

### 3.3. Core Dataset Processing Workflow for Chu-Style Lacquerware

In terms of data processing, this stage is a crucial step in constructing high-quality images suitable for training deep learning models [32]. Online resources often suffer from coarse annotations and insufficient image clarity, which hinder the ability of models to learn the pattern features of Chu-style lacquerware. Such problems are easily amplified during large-scale model training and may even lead to model collapse [33]. where the artistic features of Chu-style lacquerware are assimilated into generic decorative styles, resulting in generated images that cannot be recognized as Chu-style lacquerware.

**Figure 4:**
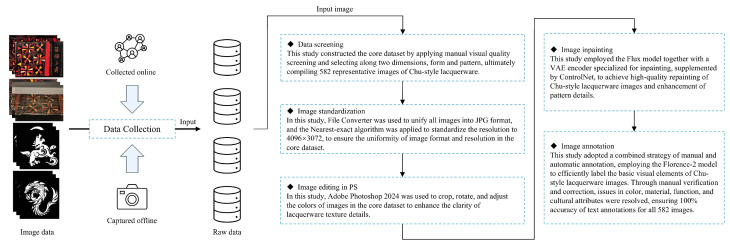
Data Processing Workflow.

Images obtained through field photography are affected by differences in equipment and shooting environments, leading to inconsistencies in format, resolution, and color space. Moreover, factors, e.g., uneven lighting, reflections from display cases, shooting angle deviations, focus errors, and shaking may further reduce image clarity and overall stability. To address these issues, the data processing pipeline of this study was designed as follows:

- Initial screening: Remove low-quality images and apply strict selection criteria for both artifact forms and decorative patterns.
- Standardization: Normalize the format and resolution of the images.
- Editing and correction: Import the screened images into Adobe Photoshop 2024 for cropping, geometric rotation, and corrections of color and lighting.
- Image refinement: Perform image redrawing to enhance overall quality and enrich pattern details.
- Annotation: Apply a combined annotation approach using automatic labeling and manual verification to ensure the quality of textual annotations. The data annotation process is illustrated in Figure 4.

#### 3.3.1. Data Screening

During the data filtering process, this study first applied manual visual assessment to the collected raw images for preliminary screening, removing invalid samples with clear quality defects, e.g., significant defocus or severe occlusion. The filtering criteria included images with blur levels exceeding a preset threshold and cases where the occlusion area of key feature regions exceeded 30%. On this basis, selection was further refined along two dimensions: vessel form and decorative pattern. At the vessel form level, representative categories, e.g., ear cups, lacquered boxes, and lacquer trays were included to ensure morphological representativeness. At the pattern level, typical Chu-style lacquerware motifs, e.g., geometric patterns, animal patterns, and cloud patterns were covered to ensure diversity of decorative features. After multiple rounds of screening and optimization, a total of 582 Chu-style lacquerware images with typical characteristics were selected to form the core dataset.

#### 3.3.2. Image Standardization

In the process of data filtering, it was found that the raw images existed in multiple storage formats, including JPG, PNG, and HEIC. To ensure the consistency of data formats, this study employed the batch conversion tool File Converter to unify all images into JPG format. Under the premise of preserving image quality, the JPG format has higher compression efficiency; at the same time, it is widely supported by mainstream deep learning frameworks, e.g., Transformer; and a unified format also helps simplify subsequent data processing workflows. File Converter v2.1 [34] is an open-source project initiated by individual developer Tichau in 2014. It is a simple and easy-to-use tool that, through the context menu of Windows File Explorer, provides users with file conversion and compression, and supports the conversion of multiple image formats including JPG, PNG, and HEIC. During the conversion process, it retains key metadata and relies on its integrated ImageMagick [35] tool to ensure overall image quality.

Due to differences in shooting equipment, the images captured during on-site photography exhibited inconsistencies in resolution. Although models can adapt to a certain degree of resolution variation during training, in order to ensure the high quality and reusability of the dataset, this study implemented resolution standardization of the images. A resolution of 4096 × 3072 pixels was chosen as the standard resolution for the CSL core dataset. This choice was based on the following three reasons:

- This resolution can fully preserve the carved details and painted strokes of Chu-style lacquerware patterns.
- It conforms to the 4:3 aspect ratio requirements of high-resolution display devices.
- While ensuring image quality, it avoids the unnecessary storage burden caused by excessively high resolutions.

**Figure 5:**
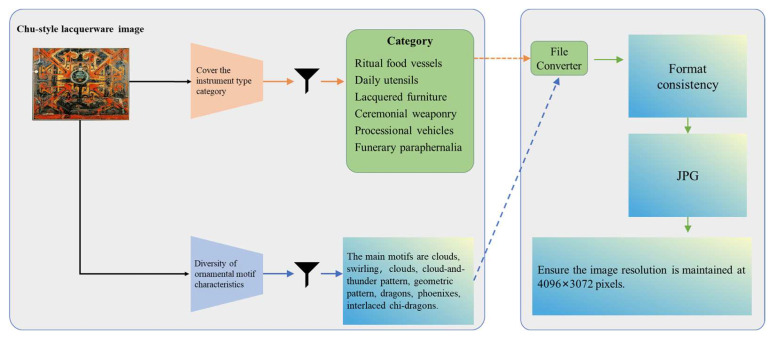
Image selection and standardization of format and resolution.

In this process, the nearest-neighbor resampling algorithm was adopted to unify image resolution. This algorithm eliminates color blending artifacts caused by interpolation, thereby achieving precise reproduction of Chu-style lacquerware patterns. The second step of the data processing workflow is shown in Figure 5.

#### 3.3.3. Image Editing and Adjustment in Adobe Photoshop

**Figure 6:**
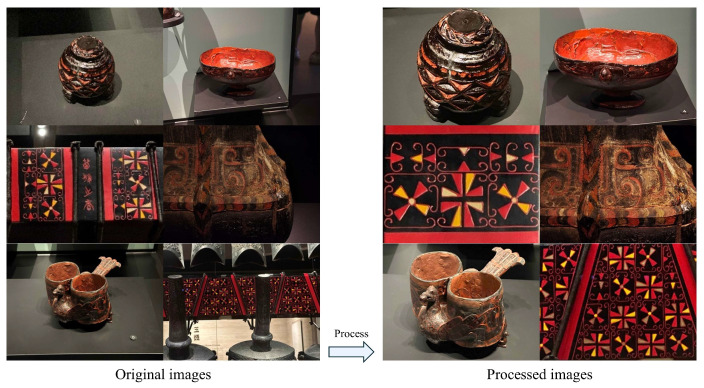
Before and after Adobe Photoshop comparison.

In the process of image editing and adjustment, addressing problems, e.g., unclear patterns, shooting angle deviations, and off-centered artifacts caused by shooting conditions at the Hubei Provincial Museum, this study imported the relevant images into Adobe Photoshop 2024 for processing, including cropping, geometric rotation, and color and lighting correction. Cropping was applied to remove interfering elements, e.g., display stands and other exhibits, ensuring the uniqueness of the Chu-style lacquerware subject within the image; geometric rotation was used to correct shooting angles, keeping the subject as centrally symmetrical as possible; color correction was performed by adjusting parameters, e.g., contrast, brightness, and exposure to improve lighting effects, thereby presenting the lacquerware’s decorative details more clearly. A comparison of images before and after processing is shown in Figure 6.

#### 3.3.4. Image Inpainting

In the image redrawing process, after the above steps were completed, an additional stage was introduced to further improve image quality and enhance the details of Chu-style lacquerware patterns. This method employs Flux as the redrawing model, using a VAE encoder specifically designed for image inpainting. The encoder encodes the original image and generates new noise maps in the target regions, which are then decoded to achieve high-quality image reconstruction. Compared with traditional VAE encoders for general image processing, this encoder is particularly optimized for image inpainting functions.

**Figure 7:**
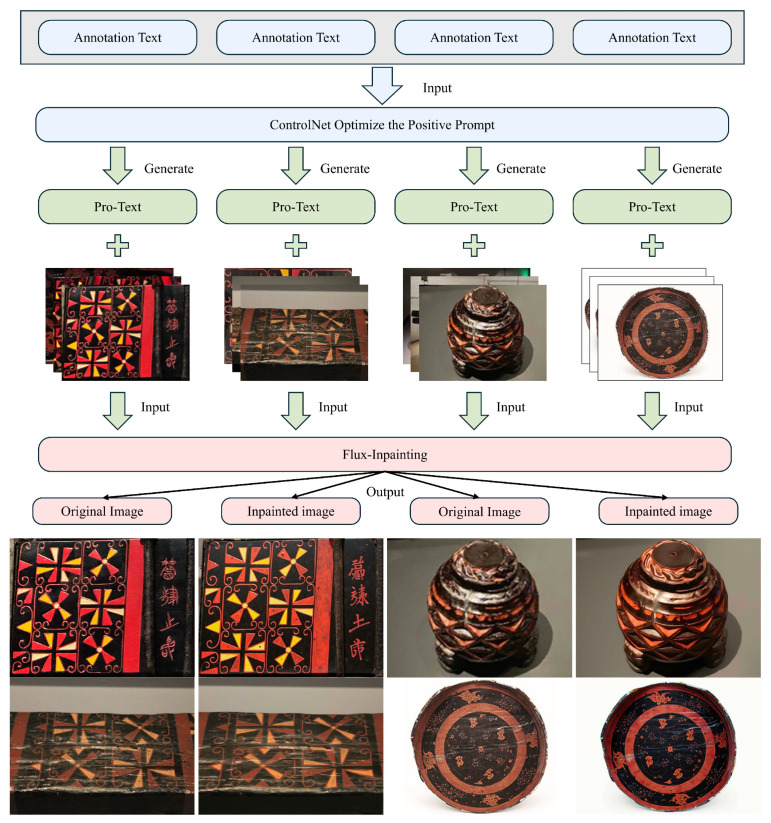
The inpainting process and comparison of the image.

During implementation, to ensure consistency between the original and redrawn images, a ControlNet model [36] was inserted between the forward prompts and the Flux model. The forward prompts were then guided through a style-control node into the Flux model. The ControlNet model adopts a synthetic complex degradation scheme, taking real images as input and applying artificial degradation through simulated noise, blurring, and JPEG compression. This significantly enhances the ability to reconstruct fine image details. The overall process is illustrated in Figure 7.

It should be specifically noted that the forward prompts mentioned in this process are essentially a form of automatic annotation text used solely for image redrawing. These do not constitute the formal textual annotations, which will be discussed in detail in the next step.

To verify the image quality of the CSL dataset, this study conducted an empirical investigation. A pre-test and post-test questionnaire design was adopted, covering four dimensions: (1) respondent background, (2) potential attractiveness, (3) technology acceptance, (4) perceived restoration effectiveness. Quantitative analysis was carried out on the survey data from 138 respondents. The results showed that respondents gave positive feedback on cultural awareness and acceptance, further demonstrating the usability of the CSL dataset.

#### 3.3.5. Image Annotation

**Figure 8:**
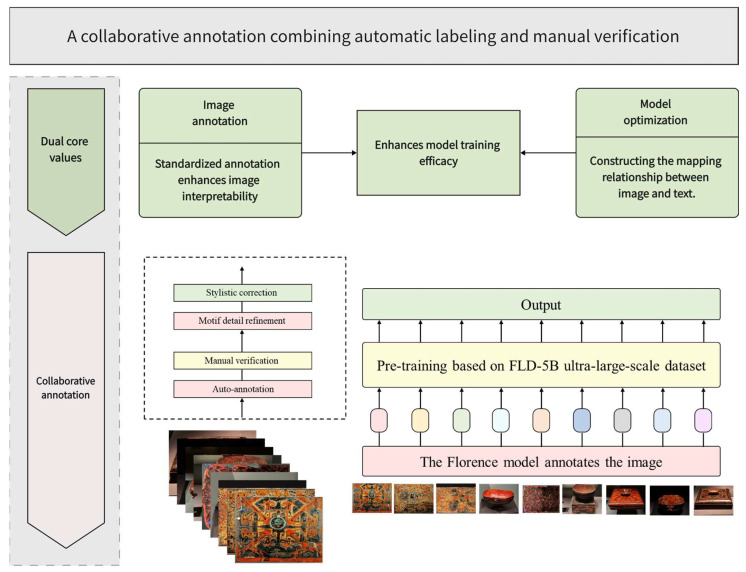
The process of text annotation.

In the image annotation process, textual annotation holds dual core value in the training of artificial intelligence generative models. At the image annotation level, standardized annotation can significantly enhance the interpretability of image content and construct effective semantic indexing for images. At the model optimization level, precise textual annotation can effectively establish the mapping relationship between textual features and image features. In this process, this study adopted a combined approach of traditional manual annotation and automatic annotation to achieve complementary advantages. Manual annotation can accurately describe the patterns of Chu-style lacquerware, but it is time-consuming, and for large-scale datasets the cycle may last several months. Automatic annotation [37] is efficient but can only identify basic visual elements and struggles to capture artistic details. By combining the two approaches and introducing manual verification, efficiency can be maintained while correcting deviations in pattern details and stylistic features. This strategy ensures that textual annotation meets both the standardization requirements of prompt engineering [38] in AI generative models and the rigor required for describing the intrinsic characteristics of cultural artifacts. Figure 8 describes the process of text annotation.

This study employed the Florence-2 (which is available at https://huggingface.co/microsoft/Florence-2-base accessed on 3 June 2025) model for automatic image annotation. The model was proposed by Microsoft Research [39] at CVPR 2024 with the aim of building a unified vision foundation model. Unlike traditional computer vision methods that rely on task-specific structures, Florence-2 adopts a prompt-based sequence-to-sequence architecture, capable of simultaneously performing multiple vision and vision-language tasks, e.g., image captioning, object detection, and semantic segmentation within a single framework. Its training relies on the large-scale FLD-5B dataset, which contains approximately 126 million images and 5.4 billion annotations, covering multi-level information at the image, region, and pixel levels. This significantly improves the model’s cross-task generalization capability. Compared with traditional vision models, Florence-2 not only exhibits stronger zero-shot and few-shot adaptability, but also comprehensively represents image semantics, regions, and pixel-level features within a unified architecture. Therefore, it is particularly suitable in this study for annotating the basic visual elements of Chu-style lacquerware images.

During manual verification, although Florence-2 was able to describe basic visual elements of the images, it exhibited limitations in describing aspects, e.g., the color, material, function, and cultural attributes of the lacquerware artifacts. For example, it occasionally misclassified gray as white in terms of color, misidentified wooden materials as metal, misinterpreted non-ritual lacquerware as ritual artifacts in terms of function, and even confused Chu-style lacquerware culture with Ancient Roman culture in cultural attributes. Nearly every image annotation contained one or more of these problems. Consequently, all 582 image annotations were examined and the incorrect parts were corrected, ensuring that the textual annotations of the dataset achieved 100% accuracy.

## 4. Methods

During dataset validation, this study will employ three base models: (1) Flux [40], (2) Stable Diffusion XL (SDXL), (3) Stable Diffusion v1.5 (SD_V1.5) [41]. In addition, the LiblibAI platform and the Segment Anything Model (SAM) will be used for the validation of the CSL dataset. A brief introduction to these models and the platform is provided below.

The Flux (which is available at https://huggingface.co/Kijai/flux-fp8 accessed on 28 June 2025) model is a series of text-to-image models developed by Black Forest Labs in Freiburg, Germany. The laboratory was founded by several of the original researchers behind Stable Diffusion, and Flux was first publicly released in August 2024. Flux adopts a Rectified Flow Transformer architecture with approximately 12 billion parameters and is trained using techniques, e.g., guided distillation, enabling a more efficient mapping from noise to data. Compared with traditional diffusion models, Flux significantly improves the quality of high-resolution text-to-image generation. The model is primarily designed for synthesizing high-fidelity images from natural language, while also supporting tasks, e.g., image editing and style transfer.

The SDXL (which is available at https://huggingface.co/stabilityai/stable-diffusion-xl-base-1.0 accessed on 3 June 2025) model was proposed by Podell et al. and published at ICLR 2024. Building upon earlier versions of Stable Diffusion, SDXL significantly expands its architecture, featuring a larger UNet backbone and dual text encoders to improve high-resolution text-to-image generation. By incorporating size conditioning, cropping conditioning, and multi-aspect ratio training, SDXL adapts more effectively to varying resolutions and image formats. The model also integrates an improved autoencoder and refinement modules to enhance local details and overall visual fidelity. Its design objective is to provide an open-source, reproducible system that delivers image generation quality competitive with black-box models.

SD_V1.5 (which is available at https://github.com/CompVis/latent-diffusion accessed on 3 June 2025), proposed by Rombach et al. and presented at CVPR 2022, is based on Latent Diffusion Models. By performing the diffusion process in latent space rather than pixel space, it significantly reduces computational costs while maintaining high-resolution image quality. Its development was aimed at achieving efficient and reproducible text-to-image generation and promoting the broader adoption of related research and applications.

LibLibAI (the official website of LibLibAI is https://www.liblib.art/ accessed on 9 June 2025) is a major Chinese platform for AI-based art generation and model sharing, hosting over 100,000 resources for image generation models across diverse styles and domains. It also supports online LoRA model training. In this study, the LiblibAI platform was used for CSL dataset validation, including model training and image generation. The platform offers a LoRA training module and an online generation module. In the LoRA training module, users can upload annotated datasets, select base models, and initiate training, with optional parameter tuning through a professional configuration interface. In the online generation module, users can perform either text-to-image or image-to-image generation. For text-to-image tasks, users can select a base model and self-trained LoRA models, configure parameters, e.g., sampling method, iteration steps, and image size, and input prompts to generate images. For image-to-image tasks, users can upload an original image, adjust parameters, and combine base models with self-trained LoRA models for generation. All images generated in this study through LiblibAI were stored locally for subsequent comparative evaluation, thereby validating the effectiveness of the CSL dataset.

The SAM (which is available at https://github.com/facebookresearch/segment-anything accessed on 28 June 2025) model, developed by Meta AI, is a foundation model for image segmentation. SAM consists of an image encoder, a prompt encoder, and a mask decoder, enabling it to generate segmentation outputs based on prompts, e.g., points, bounding boxes, or text. Trained on the SA-1B dataset containing 11 million images and 1.1 billion masks, SAM demonstrates strong cross-task and cross-domain generalization, supporting a wide range of segmentation scenarios without additional training.

The validation methodology is as follows:

- For the CSL core dataset, validation was conducted from four perspectives: (1) text-to-image generation, (2) image-to-image generation, (3) multi-resolution generation, (4) image segmentation.
- For the CSL style dataset, validation was conducted through text-to-image generation experiments.

**Figure 9:**
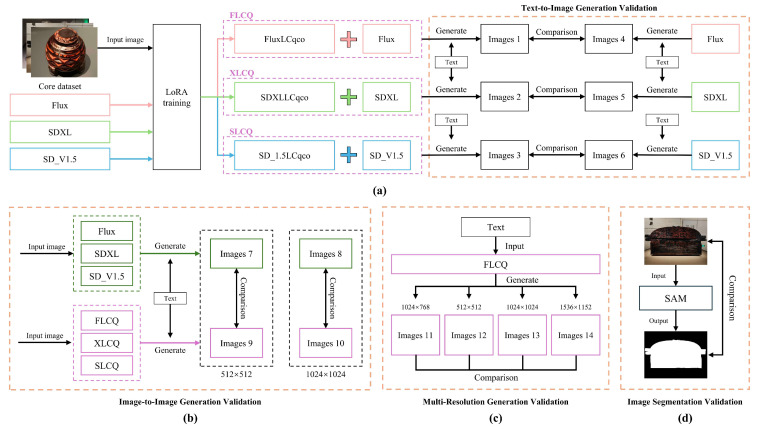
The validation methods of the CSL core dataset are illustrated as follows: (**a**) validation of text-to-image generation, (**b**) validation of image-to-image generation, (**c**) validation of multi-resolution generation, and (**d**) validation of image segmentation.

Figure 9 illustrates the validation method for the core dataset.

The first validation dimension for the CSL core dataset was text-to-image generation. In this experiment, the study applied the LoRA technique to fine-tune the three base models (Flux, SDXL, and SD_V1.5) using the CSL core dataset. The resulting LoRA models were named FluxLCqco, SDXLLCqco, and SD_1.5Lcqco, respectively. Since these fine-tuned models require their corresponding base models to operate, a naming convention was adopted to distinguish different generation methods: (1) when FluxLCqco is used with Flux, the method is termed FLCQ; (2) when SDXLLCqco is used with SDXL, the method is termed XLCQ; (3) when SD_1.5LCqco is used with SD_V1.5, the method is termed SLCQ. Table 6 provides an overview of image generation methods combining LoRA with base models.

Following the fine-tuning of these models, two sets of image generation experiments were conducted. The first set employed Flux, SDXL, and SD_V1.5 directly, while the second set used the FLCQ, XLCQ, and SLCQ methods. The generated images were compared, and their quality was evaluated along three dimensions: (1) overall stylistic consistency, (2) structural fidelity of lacquerware forms, (3) detail generation of patterns. If the images generated by the second set demonstrated higher quality, it would serve as evidence of the CSL core dataset’s effectiveness.

The second validation dimension of the CSL core dataset is image-to-image generation. In this experiment, two images, three base models (Flux, SDXL, and SD_V1.5), and three generation methods (FLCQ, XLCQ, and SLCQ) were employed to validate the effectiveness of the CSL core dataset at two resolutions: 512 × 512 and 1024 × 1024. At each resolution, two sets of image generation were conducted, resulting in a total of four sets. At both resolutions, the first set utilized Flux, SDXL, and SD_V1.5 for generation, while the second set employed the FLCQ, XLCQ, and SLCQ methods. After generation, the two sets of images at each resolution were compared, and the quality of the generated images was evaluated from three dimensions: (1) Overall Style (OS), (2) Line Features (LF), (3) Curvature Features (CF). If the second set of generated images demonstrated higher quality at both resolutions, it would validate the effectiveness of the CSL core dataset. Table 7 defines the three evaluation dimensions and their corresponding criteria.

The third validation dimension of the CSL core dataset is multi-resolution generation. In this experiment, the FLCQ method was employed to generate one set of images at each of four resolutions: 512 × 512, 1024 × 768, 1024 × 1024, and 1536 × 1152, with each set consisting of four images. Upon completion, the generated images were comparatively evaluated across resolutions from two perspectives: overall style and pattern details. The objective was to determine the optimal resolution for fine-tuning Flux with the CSL core dataset and to further verify the dataset’s usability across different resolutions.

The fourth validation dimension of the CSL core dataset is image segmentation. In this experiment, the SAM was used to assess the dataset’s quality validation [42,43]. The evaluation was based on the alignment between generated masks and the structural contours of artifacts. If the generated masks accurately matched the structural contours, it would confirm the effectiveness of the CSL dataset.

**Figure 10:**
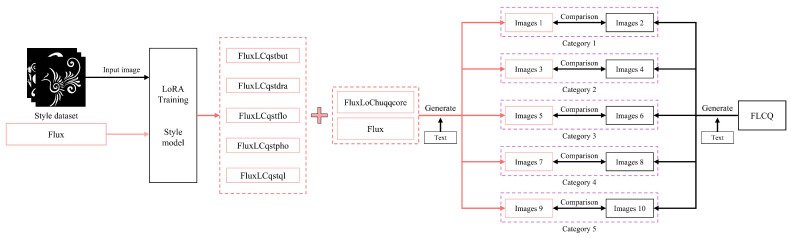
Validation method of the style dataset.

The validation of the CSL style dataset was conducted in the domain of text-to-image generation. Figure 10 illustrates the validation method for the style dataset. In this study, LoRA was applied to the CSL core dataset to fine-tune Flux, SDXL, and SD_V1.5, resulting in three core models. Additionally, five style models were fine-tuned using the CSL style dataset: (1) FluxLCqstbut, (2) FluxLCqstdra, (3) FluxLCqstflo, (4) FluxLCqstpho, (5) FluxLCqstql. For clarity, models trained on the CSL core dataset are referred to as core models, whose objective is to generate images that align with the morphological and pattern features of Chu-style lacquerware; models trained on the CSL style dataset are referred to as style models, whose objective is to generate innovative Chu-style lacquerware patterns. The five style models were designed for different types of patterns: (1) FluxLCqstbut for the *Butterfly Patterns*, (2) FluxLCqstdra for the *Dragon Patterns*, (3) FluxLCqstflo for the *Floral Patterns*, (4) FluxLCqstpho for the *Phoenix Patterns*, (5) FluxLCqstql for the *Qilin Patterns*. Table 8 Classification and Functional Description of Models Based on the CSL Dataset.

The style models cover five categories of innovative patterns: (1) butterfly, (2) dragon, (3) floral, (4) phoenix, (5) qilin. Based on these categories, five types of prompts were constructed, and the FLCQ method was used to generate five corresponding sets of images. Building on the FLCQ method, each of the five style models was then incorporated, and the corresponding category-specific prompts were applied to generate another five sets of images. Upon completion, the generated results were compared by prompt category between the baseline FLCQ method and the FLCQ method augmented with style models. If, across the five prompt categories, the latter demonstrated superior performance in generating innovative patterns, it would validate the effectiveness of the CSL style datas.

## 5. Technical Validation

### 5.1. Validation Results

**Figure 11:**
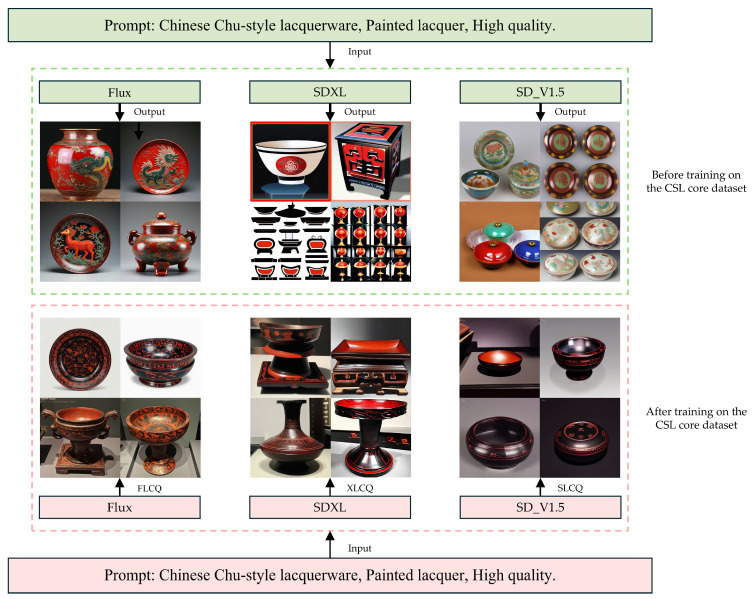
Verification results of text-to-image generation.

The validation results of text-to-image generation on the CSL Core Dataset are illustrated in Figure 11. In this study, three base models, Flux, SDXL, and SD_V1.5, were trained on the CSL Core Dataset, and their generated results were comparatively analyzed. The experiments indicate that models fine-tuned on the CSL core dataset align more closely with the stylistic characteristics of Chu-style lacquerware: they not only reproduce typical morphological features of the vessels but also present finer lacquerware Patterns on the surfaces, showing significant improvements over untuned baselines and thereby confirming the effectiveness of the CSL dataset. To further examine effectiveness across base models, we conducted a cross-method comparison among FLCQ, XLCQ, and SLCQ. All three methods preserved the overall stylistic consistency of Chu-style lacquerware, but they differed in morphology and Pattern rendition: (1) SLCQ tended toward planar depictions and lacked realism; (2) XLCQ improved morphology but only modestly; (3) FLCQ achieved the best morphological fidelity and Pattern detail, yielding images that are more realistic, natural, and esthetically refined. Overall, the CSL core dataset proved most effective with Flux. A comparative summary of text-to-image models fine-tuned on the CSL core dataset is provided in Table 9.

**Figure 12:**
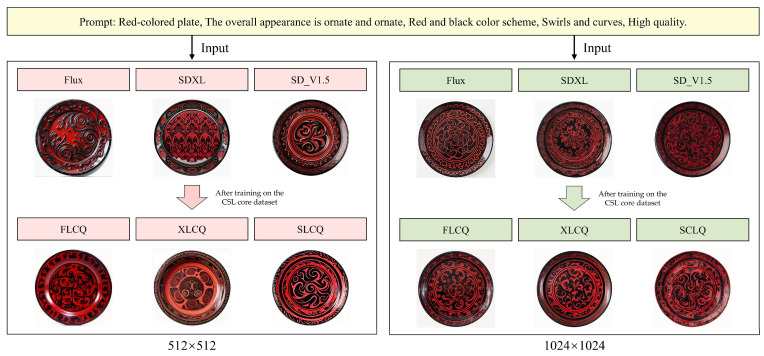
Verification results of image-to-image generation.

**Figure 13:**
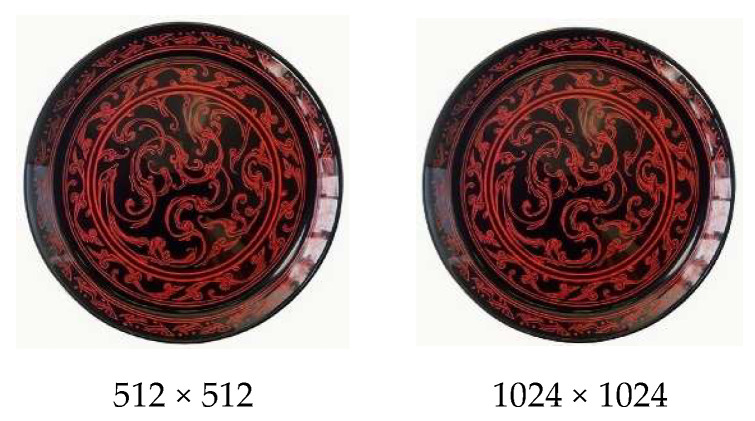
Display of the original image in image-to-image generation.

The validation results of the image-to-image generation capability of the CSL core dataset are shown in Figure 12, while Figure 13 presents the original images at resolutions of 512 × 512 and 1024 × 1024. As summarized in Table 10, the comparison highlights the performance of models before and after training.

At the 512 × 512 resolution, the untrained Flux, SDXL, and SD_V1.5 models all deviated from Chu-style lacquerware standards in the OS dimension and generally failed to exhibit Chu-style features. The Flux model generated images mainly consisting of line-based patterns (LF = Yes), with some lines showing curvature similar to the original image, while others were excessively curved (CF = Yes). In contrast, the SDXL and SD_V1.5 models produced images without line-based patterns (LF = No), and CF was not preserved. After training with the CSL core dataset, evaluations of images generated by FLCQ, XLCQ, and SLCQ showed improvements in OS across all three methods. FLCQ preserved Chu-style stylistic features, generated line-based patterns, and produced more lines with curvature similar to the original image. XLCQ and SLCQ also preserved Chu-style features and generated line-based patterns, with XLCQ still producing excessively curved lines, whereas SLCQ generated lines with curvature more closely resembling the original image.

At the 1024 × 1024 resolution, the untrained models generally maintained Chu-style features in OS, but their performance varied in LF and CF. The Flux model generated line-based patterns (LF = Yes), but the lines were excessively curved (CF = No). The SDXL model produced non-line-based patterns (LF = No) and failed to preserve CF. The SD_V1.5 model generated line-based patterns (LF = Yes) and partially preserved CF. After training with the CSL core dataset, FLCQ, XLCQ, and SLCQ all demonstrated further improvements in OS and CF. FLCQ preserved line-based patterns while enhancing both OS and CF. XLCQ showed improvements across all three dimensions (OS, LF, and CF). SLCQ preserved LF while also improving OS and CF. Table 10 summarizes the performance of the three base models, Flux, SDXL, and SD_V1.5, before and after training with the CSL core dataset, evaluated at both 512 × 512 and 1024 × 1024 resolutions. The evaluation dimensions include OS, LF, and CF, with “Enhancement Effect” indicating the dimensions that showed improvement after training.

**Figure 14:**
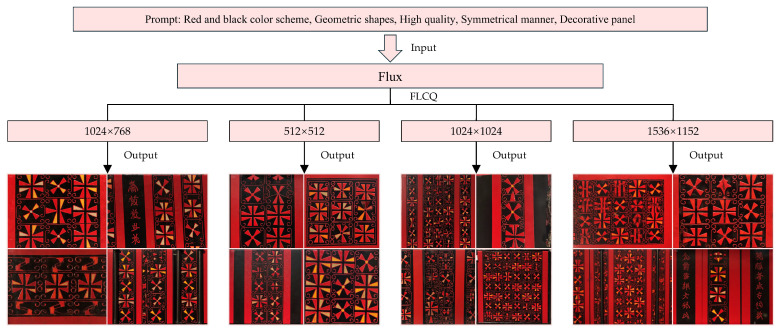
Verification results of multi-resolution generation capability.

**Figure 15:**
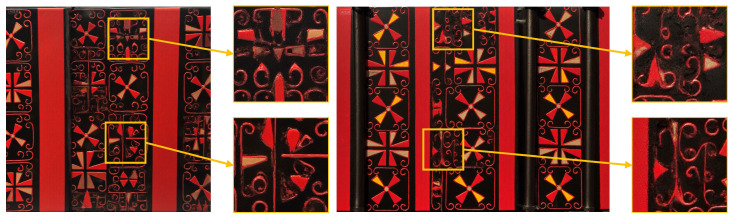
Texture errors cases.

The multi-resolution generation validation results of the CSL core dataset are shown in Figure 14. The experiments demonstrate that images generated at all four resolutions maintained the stylistic characteristics of Chu-style lacquerware without significant deviation, thereby confirming the effectiveness of the CSL core dataset. In terms of Pattern details, the 512 × 512 resolution yielded the highest image quality, with no Pattern errors observed. At 1024 × 768 and 1024 × 1024 resolutions, the OS was preserved, but varying degrees of Pattern errors occurred. At the higher resolution of 1536 × 1152, Pattern errors became more pronounced. Therefore, the 512 × 512 resolution produced the best generative results and can be regarded as the optimal resolution for fine-tuning Flux based on the CSL core dataset. Figure 15 presents typical cases of Pattern errors.

**Figure 16:**
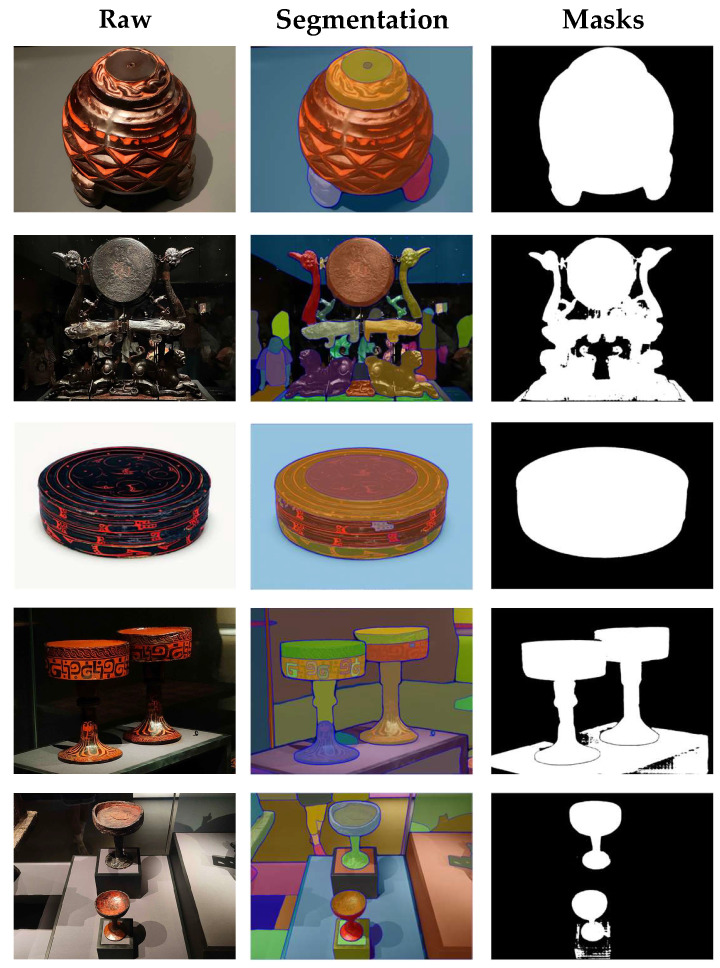
Quality validation result of the dataset.

The image segmentation validation results of the CSL core dataset are shown in Figure 16. Using the SAM developed by Meta AI for segmentation, the results demonstrate that the generated masks were highly consistent with the structural contours of artifacts in both overall shape and local details, further confirming the usability of the CSL core dataset.

**Figure 17:**
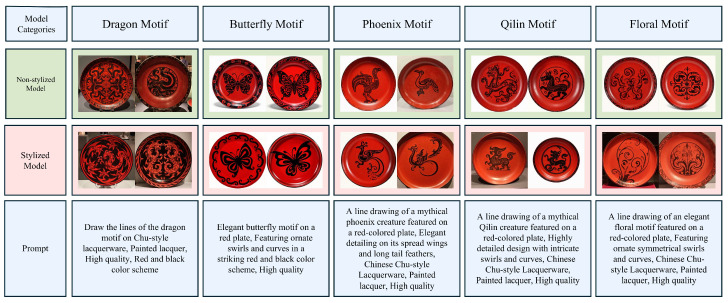
Validation Results of Style Fusion Capability.

The text-to-image validation results of the CSL style dataset are shown in Figure 17. The experimental results indicate that without incorporating style models, the *Dragon Pattern* appeared as chaotic lines, the *Butterfly Pattern* was overly realistic, the *Phoenix Pattern* lacked crown and tail-feather features and resembled an ordinary bird, the *Qilin Pattern* was incorrectly generated as a generic animal form, and the *Floral Pattern* failed to capture detailed plant motifs. After incorporating style models, the *Dragon Pattern* employed abstract lines to enhance its majestic imagery, the *Butterfly Pattern* presented simplified yet decorative line features, the *Phoenix Pattern* accurately highlighted the crown and tail feathers, the *Qilin Pattern* restored the auspicious beast imagery described in classical texts, and the *Floral Pattern* fully expressed the structural esthetics of plant motifs. In summary, the FLCQ method augmented with style models outperformed the baseline FLCQ method in generating innovative Patterns, thereby validating the effectiveness of the CSL style dataset.

### 5.2. Discussion

A comprehensive analysis of the experimental results indicates that the CSL core dataset exhibited strong applicability and effectiveness in both image generation and segmentation tasks. In text-to-image and image-to-image tasks, models fine-tuned with the CSL core dataset surpassed untrained models in stylistic consistency, morphological fidelity, and Pattern detail generation, proving the dataset’s capacity to enhance generative model performance. Among the tested methods, FLCQ achieved the best performance in morphological realism and Pattern naturalness, highlighting the stronger compatibility between Flux and the CSL core dataset. In the multi-resolution experiments, the best performance in both stylistic consistency and Pattern detail was achieved at 512 × 512 resolution, while higher resolutions aggravated Pattern errors, indicating greater practicality of the CSL core dataset under low- and mid-resolution conditions. In the segmentation validation, masks generated by the SAM closely matched artifact structural contours, confirming the accuracy and reliability of CSL core dataset annotations at the structural level.

The results of the CSL style dataset demonstrated that style models effectively compensated for the shortcomings of FLCQ in generating innovative Patterns. The generated results not only maintained Chu-style lacquerware esthetics but also exhibited diversity and creativity in Patterns. This validated the effectiveness of the CSL style dataset and underscored its potential value for the digital reproduction and innovative design of Chu-style lacquerware.

Overall, the CSL core dataset and style dataset are complementary. The former plays a critical role in ensuring the authenticity and consistency of Chu-style lacquerware morphology and Patterns, while the latter supports stylistic diversity and innovative expression. Together, they not only demonstrate the academic and practical value of the CSL dataset but also lay a foundation for future applications in the digital preservation and inheritance of intangible cultural heritage and in artistic style generation research.

## 6. Conclusions and Future Work

### 6.1. Conclusions

This study focuses on the digital preservation and inheritance of Chu-style lacquerware by constructing the first Chu-style Lacquerware Dataset (CSL Dataset) specifically designed for AI generative models. The dataset systematically integrates on-site photographic resources and official digital archives from the Hubei Provincial Museum, comprising 582 high-resolution core images, 37 style pattern images, and 72 videos of artifacts, thereby providing a relatively comprehensive representation of the vessel forms and pattern characteristics of Chu-style lacquerware.

The usability of the CSL core dataset was verified across four tasks: text-to-image generation, image-to-image generation, multi-resolution generation, and image segmentation. The CSL style dataset was validated through text-to-image generation experiments. The results indicate that the CSL core dataset significantly enhances model performance in terms of overall stylistic consistency, structural fidelity, and detail generation of patterns, among which the Flux-based FLCQ method yielded the best results. The dataset maintained stable style characteristics across different resolutions, with the most natural and consistent generative performance observed at 512 × 512 resolution. Segmentation experiments using the SAM demonstrated that the generated masks aligned closely with the structural contours of artifacts, further confirming the usability of the CSL core dataset. Meanwhile, the CSL style dataset effectively compensated for the limitations of single-generation approaches in innovative pattern creation, optimizing the generative effect of traditional Chinese patterns and validating its applicability in producing novel Chu-style lacquerware patterns.

Together, the CSL core and style datasets complement each other, not only verifying their effectiveness and application value in the digital study of Chu-style lacquerware but also providing a new technological pathway and methodological support for the digital preservation and inheritance of ICH.

### 6.2. Future Work

The interdisciplinary CSL dataset can assist researchers in the arts in conducting in-depth analyses of Chu-style lacquerware pattern features, form evolution, and cultural connotations, providing visual data support for art historical research. It can also support researchers in computer vision in developing high-precision image generation and restoration algorithms, advancing the application of generative AI in the cultural heritage field. Future studies may build on the CSL dataset to further explore more refined image style transfer techniques, enabling precise capture and reproduction of artistic styles from different historical periods of Chu-style lacquerware, as well as to investigate innovative models of human–AI collaboration that integrate AI generation with traditional craft knowledge for applications in artifact restoration and creative design practices.

## Figures and Tables

**Table 1:** Overview of Safeguarding Techniques for ICH Handicrafts.

Author (Year)	Protection Path	Protection Object	Related Disciplinary Direction
Marek Milosz et al. (2022) [12]	3D Technology	Intangible Cultural Heritage	Computer Science
Huafeng Quan et al. (2024) [13]	Knowledge Graph, Natural Language Processing, Deep Learning Techniques	Miao batik culture in Guizhou Province, China	Interdisciplinary field of Computer Science and Design
Ying Huang et al. (2024) [14]	Distributed Optical Fiber Sensor Corrosion Monitoring Technology	Handicraft using steel as material	Interdisciplinary field of Materials Science and Optics
Zishan Xu et al. (2024) [15]	Dataset protection	Dunhuang murals	Interdisciplinary field of Computer Science and Design

**Table 2:** Description of the Current State of Lacquerware Research.

Author (Year)	Research Object	Region	The Language Used for Writing the Paper	Whether to Synchronize the Lacquerware Images
Liping Zheng et al. (2020) [16]	Lacquerware from the Late Qing period	Bashu area, China	English	**×**
Yingchun Fu et al. (2020) [17]	Lacquerware unearthed from the Chu tombs at Jiuliandun, Hubei, Warring States period	Hubei, China	English	**×**
Meng Wu et al. (2021) [18]	Colored lacquerwares from Zenghou Yi Tomb in Early Warring States	Hubei, China	English	**×**
Xin Wang et al. (2021) [19]	lacquered wooden coffins from Eastern Regius Tombs of the Qing Dynasty	Hebei, China	English	**×**
Bin Han et al. (2022) [20]	The Warring States lacquer scabbard unearthed from the Lijiaba site	Chongqing, China	English	**×**
Kai Wang et al. (2022) [21]	Ancient lacquer films of the Western Han Dynasty	Shanxi, China	English	**×**
Danfeng Hu et al. (2023) [22]	Lacquer fragments salvaged from the “Nanhai I” shipwreck	Guangdong, China	Chinese	**×**
Zisang Gong et al. (2024) [23]	Painted gold foils applied to lacquerware of the Late Western Han Dynasty	Shanxi, China	English	**×**
Zhanyun Zhu et al. (2025) [24]	Lacquer screen of the Northern Wei period	Shanxi, China	English	**×**
Jiake Chen et al. (2025) [25]	Han dynasty lacquerware unearthed from M9, Guishan, Luoping, Yunnan	Yunnan, China	Chinese	**×**

**Table 3:** Dataset Introduction.

Dataset Classification	Type	Count	Resolution
Core Dataset	Image (.jpg)	582	4096 × 3072
	Text (.txt)	582	
Style Dataset	Image (.jpg)	37	1024 × 1024
	Text (.txt)	37	
Video Dataset	Video (.mov + .mp4)	72 (43.mov + 29.mp4)	1920 × 1080

**Table 4:** Dataset Comparison.

Data Source	Image Count	Resolution	Dataset Description	Data Composition	Cultural Attribute Depth	Data Reliability
Heluo Musical Relics_Xia-Qing [26]	102	400 × 300	Research on musical relics created and preserved from Xia-Shang to Ming-Qing dynasties	Typological classification, cross-cultural terminology	Typological evolution lineage of musical relics	Academically consensual terminology
Yungang Grottoes [27]	Not specified	Not specified	3D scanned point cloud image data of Cave 13 at Yungang Grottoes, Shanxi Province	3D models, high-definition images, environmental monitoring data	Religious artistic symbol interpretation	High-precision scanning
Embroidery Cultural Relics [28]	261	737 × 821 800 × 726	Embroidered costumes from the mid-late Qing Dynasty to the Republican era	Image data, statistical information tables	Embroidery technique documentation	Field-measured data
DeepJiandu [29]	7416	Width: 150–600+ pixels Height: 100–3200+ pixels	Jiandu Detection and Recognition	Infrared Imaging (BMP) VOC-Format Annotations	2242-Class Ancient Script Taxonomy	Gaussian-Filtered Denoising
Art_GenEvalGPT [30]	Not specified	Not specified	Art-Themed Dialogs Generated via ChatGPT	Dialog Corpora (CSV/JSON Formats)	26 Art Movements 378 Artist Referents	Automated Metric Evaluation Human-Validated Affective Annotation
CSL (Ours)	582 *	4096 × 3072	Collection and organization of Chu-style lacquerware images	Vessel shape and pattern images, vessel videos	The culture of Chu lacquerware	Multi-faceted validation from technology and society

* The image count statistics only include the core dataset and do not account for the style dataset and video dataset.

**Table 5:** Comparison of Advantages and Limitations of Different Methods.

Method	Advantages	Limitations	
Online Resources Collection	Strong systematization and wide coverage; provides a relatively complete classification system of Chu-style lacquerware	Some images have low resolution; blurred edges; insufficient color information, making them unsuitable for training needs	
On-site Photography	Ensures comprehensive category coverage; expands sample size within single categories; produces higher-quality images more suitable for deep learning training	Restricted by exhibition hall lighting, reflections, and shooting angles; some images may contain noise, glare, or reduced clarity	
Data Processing	Constructing high-quality images suitable for training datasets in deep learning models	Online resources	On-site images
		rough labels, unclear images; Model Collapse	Inconsistencies in format, resolution, and color space; reduced image clarity

**Table 6:** Overview of Image Generation Methods Combining LoRA with Base Models.

Model Source	Base Model	LoRA-Based Generation Method	Method Description
Black Forest Labs [40]	Flux	FLCQ	When FLCQ is combined with Flux for generation
Podell et al. [7]	SDXL	XLCQ	When XLCQ is combined with SDXL for generation
Rombach et al. [41]	SD_V1.5	SLCQ	When SLCQ is combined with SD_V1.5 for generation

**Table 7:** Evaluation Dimensions and Criteria for Image Generation Quality.

Abbreviation	Dimension	Evaluation Criteria
OS	Overall Style	Whether the overall image conforms to the Chu-style lacquerware style
LF	Line Features	Whether the main patterns are composed of lines
CF	Curvature Features	Whether the degree of line curvature is similar to the original image

**Table 8:** Classification and Functional Description of Models Based on the CSL Dataset.

Model Name	Model Type	Dataset	Base Model	Fine-Tuning Method	Application
FluxLCqco	Core Model	Core Dataset	Flux	LoRA	Generate images that conform to the morphological and pattern features of Chu-style lacquerware
SDXLLCqco	Core Model	Core Dataset	SDXL	LoRA	
SD_1.5LCqco	Core Model	Core Dataset	SD_V1.5	LoRA	
FluxLCqstbut	Style Model	Style Dataset	Flux	LoRA	Generate innovative Chu-style lacquerware patterns
FluxLCqstdra	Style Model	Style Dataset	Flux	LoRA	
FluxLCqstflo	Style Model	Style Dataset	Flux	LoRA	
FluxLCqstpho	Style Model	Style Dataset	Flux	LoRA	
FluxLCqstql	Style Model	Style Dataset	Flux	LoRA	

**Table 9:** Comparative Analysis of Text-to-Image Models Fine-Tuned on the CSL Core Dataset.

Model	Advantages	Limitations
SD_V1.5	After training on the CSL dataset, the overall style conforms to Chu-style lacquerware; capable of generating images with certain stylistic features	Morphology tends to be planar, lacking realism; Pattern details deviate from Chu-style lacquerware standards, appearing less natural and refined
SDXL	The basic morphology of artifacts is improved compared with SD_V1.5; lacquerware forms are closer to real objects	Improvements are limited; overall morphological fidelity remains insufficient; stylistic consistency and Pattern details still show deviations
Flux	Best morphological fidelity with strong image realism; optimal stylistic consistency; natural, rich, and elegant Patterns that conform to Chu-style lacquerware characteristics	No significant limitations were observed in the validation of this module

**Table 10:** Comparative Training Effects of Image Generation Models at Different Resolutions.

Resolution	Base Model	Criteria	Before Training	After Training	Enhancement Effect
512 × 512	Flux	OS	Deviated	Maintained	OS, CF
		LF	Yes	Yes	
		CF	Yes	Improved	
	SDXL	OS	Deviated	Maintained	OS, LF
		LF	No	Yes	
		CF	No	No	
	SD_V1.5	OS	Deviated	Maintained	OS, LF, CF
		LF	No	Yes	
		CF	No	Yes	
1024 × 1024	Flux	OS	Maintained	Improved	OS, CF
		LF	Yes	Yes	
		CF	No	Yes	
	SDXL	OS	Maintained	Improved	OS, LF, CF
		LF	No	Yes	
		CF	No	Yes	
	SD_V1.5	OS	Maintained	Improved	OS, CF
		LF	Yes	Yes	
		CF	Yes	Improved	

## Data Availability

CSL is publicly available on Hugging Face at https://huggingface.co/datasets/TenFate/CSL_Dataset (accessed on 2 September 2025). Core and style models developed in this study are accessible at https://huggingface.co/TenFate/CSL_Generation_Model (accessed on 2 September 2025).

## Abbreviations

The following abbreviations are used in this manuscript:

CSL	Chu-style Lacquerware Dataset
LoRA	Low-Rank Adaptation
AI	Artificial Intelligence
ICH	Intangible Cultural Heritage
SDXL	Stable Diffusion XL
SD_V1.5	Stable Diffusion v1.5
SAM	Segment Anything Model
OS	Overall Style
LF	Line Features
CF	Curvature Features
